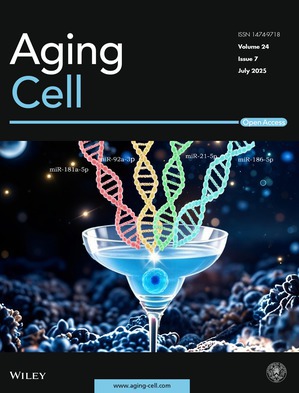# Additional Cover

**DOI:** 10.1111/acel.70173

**Published:** 2025-07-16

**Authors:** Tianpeng Zhang, Allancer D. C. Nunes, Jieun Lee, Dana Larocca, Giovanni Camussi, Sai Kiang Lim, Vicky U. Bascones, Luise Angelini, Ryan D. O'Kelly, Xiao Dong, Laura J. Niedernhofer, Paul D. Robbins

## Abstract

Cover legend: The cover image is based on the article *Identification of Senomorphic miRNAs in Embryonic Progenitor and Adult Stem Cell‐Derived Extracellular Vesicles* by Tianpeng Zhang et al., https://doi.org/10.1111/acel.70071.